# Engine oil from agricultural machinery as a source of PCDD/Fs and PCBs in free-range hens

**DOI:** 10.1007/s11356-022-24180-5

**Published:** 2022-11-23

**Authors:** Marek Pajurek, Szczepan Mikolajczyk, Malgorzata Warenik-Bany

**Affiliations:** Radiobiology Department, National Veterinary Research Institute, NRL for Halogenated POPs (PCDD/Fs, PCBs and PBDE) in Food and Feed, 57 Partyzantów Avenue, 24-100, Pulawy, Poland

**Keywords:** PCDD/Fs, PCBs, Eggs, Engine oil, Risk

## Abstract

Free-range hens spend most of their lives outdoors, resulting in their heavy exposure to environmental pollutants such as polychlorinated dibenzo-p-dioxin, dibenzofurans (PCDD/Fs), dioxin-like polychlorinated biphenyls (DL-PCBs), and non-dioxin-like polychlorinated biphenyls (NDL-PCBs). We present a case of contamination of free-range eggs that is previously unreported in the literature. The aim of our study was a source investigation after finding a high level of PCDD/Fs in samples of eggs from one of the inspected farms. Samples of hens’ eggs, muscles, and livers and the feeds and soils were analyzed. The results showed that the soil samples taken from the paddock contained high concentrations of PCDD/Fs and DL-PCBs expressed as toxic equivalents (TEQ) (72.9 ± 18.2 pg WHO-TEQ g^−1^ dry mas (d.m.)) and a high concentration of NDL-PCBs (207 ± 46.9 ng g^−1^ d.m.). The investigation found that the cause of the soil contamination was oil leaking from the farm’s tractor engine. The oil contained very high concentrations of PCDD/F and DL-PCBs (1013 ± 253 pg WHO-TEQ g^−1^ oil) and 5644 ng g^−1^ of NDL-PCBs. The source of the contamination was confirmed by the similarity of the PCDD/F and PCB profiles in the hen eggs and the soil contaminated by engine oil. The dietary intake of toxins resulting from consumption of the eggs is provided. For children, the consumption of contaminated eggs would result in an intake of double the tolerable weekly intake (TWI), while for adults, it would be approx. 60–70% of TWI.

## Introduction

Hen eggs are a fundamental part of people’s diets around the world. For many years, the main way in which hens were industrially reared was in cages. As consumers have become more aware of animal welfare issues, the demand for eggs produced in systems other than cages has increased. Hen eggs from backyard production are becoming increasingly popular among consumers, who expect that the food has high nutritional value and is free from toxic substances (Di Pillo et al. 2019; Rajkumar et al. 2021). However, because free-range hens spend most of their lives outdoors, they are much more likely to be exposed to environmental contaminants, such as PCDD/Fs and PCBs (Hsu et al. 2010; Roszko et al. 2014; Pajurek et al. 2019), heavy metals (Cowie and Gartrell 2019; Atamaleki et al. 2020; Yazdanparast et al. 2022), and perfluorinated or brominated compounds (Pajurek et al. 2019; Gazzotti et al. 2021; Mikolajczyk et al. 2022). PCDD/Fs and PCBs are toxic substances that are commonly found in the environment, belonging to the group of persistent organic pollutants (POPs) (Stockholm Convention 2001). They are characterized by long degradation times and lipophilic properties, which result in their bioaccumulation in the fatty tissue of animals and their biomagnification in the food chain (Romero-Romero et al. 2017; Castro-Jiménez et al. 2021; Prince et al. 2021). Long-term exposure of humans to these compounds, even at very low doses, can increase the risk of adverse health effects, such as hormonal imbalances, immunological disorders, reproductive disorders, and cancer (Mocarelli et al. 2008; EFSA 2013, 2018; Fábelová et al. 2019; Onozuka et al. 2021; Rocha et al. 2021). It should be pointed out that the health effects of exposure to PCDD/Fs may not become apparent until many years after the exposure and also in subsequent generations (Mocarelli et al. 2008; Baccarelli et al. 2008; Viluksela and Pohjanvirta 2019). As the primary route of exposure to humans is food of animal origin, it is very important to control the quality of animal products against contamination with PCDD/Fs and PCBs. Even though maximum levels in feed materials (Directive 277/2012/EU, 1259/2011/EU and recommendations 2013/711/EU, 2014/663/EU) have been introduced by the European Community to limit PCDD/Fs and PCBs in feed and food, incidents of contamination of food of animal origin still occur. It has been known for many years from studies that eggs from free-range hens may be a dietary source of PCDD/Fs and PCBs for consumers (Piskorska-Pliszczynska et al. 2014; Hoogenboom et al. 2016; Pajurek et al. 2019; Mikołajczyk et al. 2021). Free-range poultry are exposed to PCDD/Fs and PCBs from many sources. The increase of these contaminants in chicken tissue and eggs may be caused by, for example, contaminated feed material, improper feed preparation, building materials preserved with chlorophenols, sewage sludge used as fertilizer, or local environmental pollution (Malisch and Kotz 2014; Piskorska-Pliszczynska et al. 2016; Hoogenboom et al. 2016). However, most incidents of contamination are caused by the hens’ access to contaminated soil (Vries et al. 2006; Piskorska-Pliszczynska et al. 2014; Mikołajczyk et al. 2021). Soil is a natural reservoir for PCDD/Fs and other persistent organic pollutants that enter it from various pollution sources (Covaci et al. 2009; Fernandes et al. 2011; Piskorska-Pliszczynska et al. 2014; Shikha and Singh 2021).

Food contamination by PCDD/Fs and PCBs related to technical oils were previously reported by other authors (Tanabe et al. 1989; Soong and Ling 1997; Covaci et al. 2008). The Belgian incident, which started when contaminated transformer oil was accidentally added to animal feed, leading to food contamination in many European countries, had the greatest impact on the international economy and consumer health (Covaci et al. 2008). Previous cases of hen contamination were related to the addition of oil to feed materials or to food, while this work shows that contaminated oil can be a source of PCDD/Fs and PCBs for hens indirectly from farm machinery left in the chicken paddock.

The aim of the study was to investigate the source of contamination of a flock of hens and to perform an assessment of the potential risks resulting from the consumption of their eggs and meat.

## Materials and methods

### Sampling and sample collection

Random sampling was carried out in the framework of the Polish National Food Survey aimed at monitoring PCDD/F and PCB background levels in food of animal origin (2006/794/EC). The sampling procedure was performed in accordance with the provisions of regulations 644/2017/EC. Each year, around 30 samples of eggs from different production types are analyzed. Samples were collected from various regions of Poland by veterinary inspectors and analyzed by the National Reference Laboratory (NRL) for halogenated POPs.

The contaminated eggs come from a farm in the western part of Poland near Wrocław. Each sample of eggs comprised twelve pieces. Muscles and livers were sampled from three animals and tested individually. Five surface soil samples (0–5 cm) were taken from places showing black spots and combined into one aggregate sample.

### Analytes of interest

The analytes of interest were in accordance with European Union legislation that sets the maximum levels of PCDD/Fs and DL-PCBs in foodstuff (Regulation 1259/2011). A total of 35 congeners, including 17 congeners of 2,3,7,8 substituted PCDD/Fs, 12 congeners of DL-PCBs (PCBs: 77, 81, 105, 114, 118, 123, 126, 156, 157, 167, 169, and 189), and 6 NDL-PCBs (28, 52, 101, 138, 153, and 180), were analyzed.

### Methods

Details of the method have been described elsewhere (Piskorska-Pliszczynska et al. 2014; Pajurek et al. 2019). Briefly, the soil and feed were dried in an oven (40 °C) for 48 h., the eggs and muscles were freeze-dried, and then the samples were spiked with internal ^13^C_12_-labeled standard which contained analogs of all the analyzed congeners (25 pg ml^−1^ for PCDD/Fs and 400 pg ml^−1^ for DL-PCBs and NDL-PCBs). All standards were purchased from the Cambridge Isotope Laboratory (Andover, MA, USA) and Wellington Laboratories Inc. (Ontario, Canada). Then, the samples were extracted using accelerated solvent extraction (ASE 300, Dionex, Sunnyvale, CA, USA). The soil was extracted with toluene, the eggs with a mixture of toluene and methanol (30:70), the muscles with dichloromethane and hexane (50:50), and the feed with toluene and acetone (70:30). All the organic solvents were of suitable purity for the residue analysis and were supplied by LGC Standard (Wesel, Germany). The extract was cleaned and fractionated using column chromatography. After pre-purification on a column of acidified silica gel (22 and 44% sulfuric acid), the extract was applied into a Florisil column and then into an active carbon column. Finally, three separate fractions were obtained, one for PCDD/Fs and the other two for DL-PCBs and NDL-PCBs. Appropriately labeled recovery control standards were added to the relevant fraction (1,2,3,4 TCDD, and PCB-111). High-resolution gas chromatography coupled with high-resolution mass spectrometry (HRGC/HRMS) was used for the analyses of the PCDDs, PCDFs, and PCBs. An Ultra Trace GC gas chromatograph (Thermo Scientific, Milan, Italy) equipped with a TriPlus Autosampler (CTC Analytics AG, Zwingen, Switzerland) coupled to a DFS (Thermo Scientific, Bremen, Germany) mass spectrometer was used.

### Quality assurance and quality control

Certified reference materials (BCR-529 sandy industrial soil, Joint Research Center, Belgium; RTC-CRM 962 clay sand, Merck, Darmstadt, Germany; and BCR-607-JRC, Belgium) and blank samples were analyzed to assure quality. Successful participation in proficiency testing (PT), organized by the European Union Reference Laboratory for Halogenated Persistent Organic Pollutants in Feed and Food (Freiburg, Germany), served as an external QC.

### TEQ calculations

Results of the sum (PCDD/F, DL-PCB, and PCDD/F/DL-PCB) are expressed according to Commission Regulation (EU) 1259/2011/EU in TEQ using the toxic equivalency factors (WHO-TEF) published in 2006 (Van den Berg et al. 2006) using the following equation:

$$TEQ=\sum_{i=1}^{7}\left({PCDD}_{i}\times {TEF}_{i}\right)+\sum_{j=1}^{10}\left({PCDF}_{j}\times {TEF}_{j}\right)+\sum_{k=1}^{12}\left({PCB}_{k}\times {TEF}_{k}\right)$$

The results are given as upper bound concentrations (concentration of each non-quantified congener (below the LOQ) was replaced with the LOQ).

## Results and discussion

### Levels of PCDD/Fs and PCBs

In the framework of a national food survey to monitor background levels of PCDD/Fs and PCBs in food of animal origin, a non-compliant sample (044-MDZ) of chicken eggs was found (Table 1). Commission Recommendation 2013/711/EU with further amendments obliges to take actions to reduce or eliminate the source of contamination. To find the source, further samples from the farm were taken by a veterinary inspector. These resampled chicken eggs (sample 148-D) also contained high levels of PCDD/Fs and PCBs (Table 1). The two fodders that the animals were being fed—149-D (feed additive, bread) and 150-D (compound feed, oats and wheat)—were both compliant and contained the analyzed contaminants at the LOQ levels (Table 1). To assess the contamination status of the chicken flock, three laying hens were sampled, and muscles and livers were collected from each animal.

**Table 1:** PCDD/F, DL-PCB, and NDL-PCB concentrations in analyzed samples (mean ± uncertainty/range)

Material		PCDD/F	DL-PCB	PCDD/F/DL-PCB	NDL-PCB
		*pg WHO-TEQ g*^*−1*^* fat*			*ng g*^*−1*^* fat*
*Limits*		2.5	-	5	40
*Action levels*		1.75	1.75	-	
Eggs	044-MDZ	4.02 ± 0.66	0.65 ± 0.12	4.67 ± 1.17	3.95 ± 0.89
	148-D	3.11 ± 0.51	1.17 ± 0.22	4.27 ± 1.07	9.71 ± 2.20
	022-BN	4.20 ± 0.69	1.41 ± 0.27	5.61 ± 1.40	3.65 ± 0.83
		*pg WHO-TEQ g*^*−1*^* fat*			*ng g*^*−1*^* fat*
*Limits*		1.75	-	3	40
*Action levels*		1.25	0.75	-	
Muscles	014-BN	2.48 ± 0.41	1.37 ± 0.26	3.85 ± 0.96	6.76 ± 1.53
	016-BN	2.86 ± 0.47	1.21 ± 0.23	4.07 ± 1.02	5.43 ± 1.23
	019-BN	4.07 ± 0.67	1.40 ± 0.26	5.46 ± 1.36	4.24 ± 0.96
		*pg WHO-TEQ g*^*−1*^* fresh weight*			*ng g*^*−1*^* fresh weight*
*Limits*		0.30	-	0.5	3.0
Livers	015-BN	0.25 ± 0.04	0.14 ± 0.03	0.39 ± 0.10	0.28 ± 0.06
	017-BN	0.30 ± 0.05	0.14 ± 0.03	0.43 ± 0.11	0.22 ± 0.05
	020-BN	0.68 ± 0.11	0.16 ± 0.03	0.83 ± 0.21	0.37 ± 0.08
		*ng WHO-TEQ kg*^*−1*^* feed*			ng kg^*−*1^ feed
*Limits*		0.75	-	1.25	10
*Action levels*		0.5	0.35		
Feed	149-D	0.05 ± 0.01	0.02 ± 0.00	0.07 ± 0.02	0.04 ± 0.01
	150-D	0.05 ± 0.01	0.06 ± 0.01	0.12 ± 0.03	0.16 ± 0.03
		*pg WHO-TEQ g*^*−1*^* soil*			*ng g*^*−1*^* soil*
Soil	012-BN	5.80 ± 0.95	67.1 ± 12.7	72.9 ± 18.2	207 ± 46.9
		*pg WHO-TEQ g*^*−1*^* oil*			*ng g*^*−1*^* oil*
Oil	023-BN	62.1 ± 10.2	951 ± 179	1 013 ± 253	5 644 ± 1 279

The concentrations of PCDD/Fs and PCBs in the muscles were above the maximum limits (ML) (014-BN, 016-BN, 019-BN). In the livers, only sample 020-BN was non-compliant due to a high concentration of PCDD/Fs and the total PCDD/Fs/DL-PCBs (Table 1). The PCDD/F levels in the liver were over two times higher than in the muscles. According to Stephens et al. (1995), on exposure to PCDD/Fs, about 5–30% of the intake is transferred to the eggs, 7–54% is deposited in the animal fat, and less than 1% in the liver. Ikeda et al. suggest that ingested PCDD/Fs are stored in the fat tissue first and then excreted into the eggs (Ikeda et al. 2004).

The farm inspection carried out by the local veterinary inspector to find the source of the contamination revealed black spots on the soil surface in the chicken paddock. A sample was taken and sent for testing (012-BN), and high concentrations of PCDDs/Fs and PCBs were found. It was determined that the black spots come from a tractor engine that was sometimes left on the chicken paddock. Finally, an oil sample (023-BN) was taken from the agricultural tractor engine, and the PCDD/F, DL-PCB, and NDL-PCB concentrations were found to be very high, reaching the level of 1013 pg WHO-TEQ g^−1^ (Table 1).

Therefore, it was assumed that the presence of PCDD/Fs and PCBs in the hens’ eggs and tissues was due to the intake of these compounds from oil-contaminated soil. Soil is a natural reservoir of PCDD/Fs and other persistent organic pollutants (Covaci et al. 2009; Fernandes et al. 2011; Piskorska-Pliszczynska et al. 2014). Free-range and organic hens have access to paddocks, which gives them greater opportunity to come into contact with environmental sources of persistent organic pollutants. Studies on the effect of different husbandry systems on the bioaccumulation of selected POPs confirm that eggs from free-range hens are more contaminated with PCDD/Fs and PCBs than eggs from cage production (Pajurek et al. 2019). Elevated PCDD/Fs levels in free-range eggs have been reported from Poland, the Netherlands, and other countries (Hsu et al. 2010; Van Overmeire et al. 2011; Piskorska-Pliszczynska et al. 2016; Hoogenboom et al. 2016). In other long-lived birds such as ostriches, which have continuous access to the open-air runs, the levels of PCDD/F/DL-PCB in their eggs can be up to 75 pg WHO-TEQ g^−1^ fat (Piskorska-Pliszczynska et al. 2017b).

It can be estimated that laying hens may take up between 2 and 30 g of soil with their feed (Waegeneers et al. 2009). This means that the theoretical daily intake of PCDD/F/PCBs by hens could reach from 146 to 2187 pg TEQ, if the laying hens only take up soil from an oil-contaminated area. However, it is unknown how much of the soil taken up by the hens came from places contaminated by engine oil, and it should also be noted that the bioavailability of PCDD/Fs from contaminated soil ingested by poultry is estimated to be around 40–60% (Van Eijkeren et al. 2006).

### Patterns of PCDD/Fs and PCBs

**Fig. 1:**
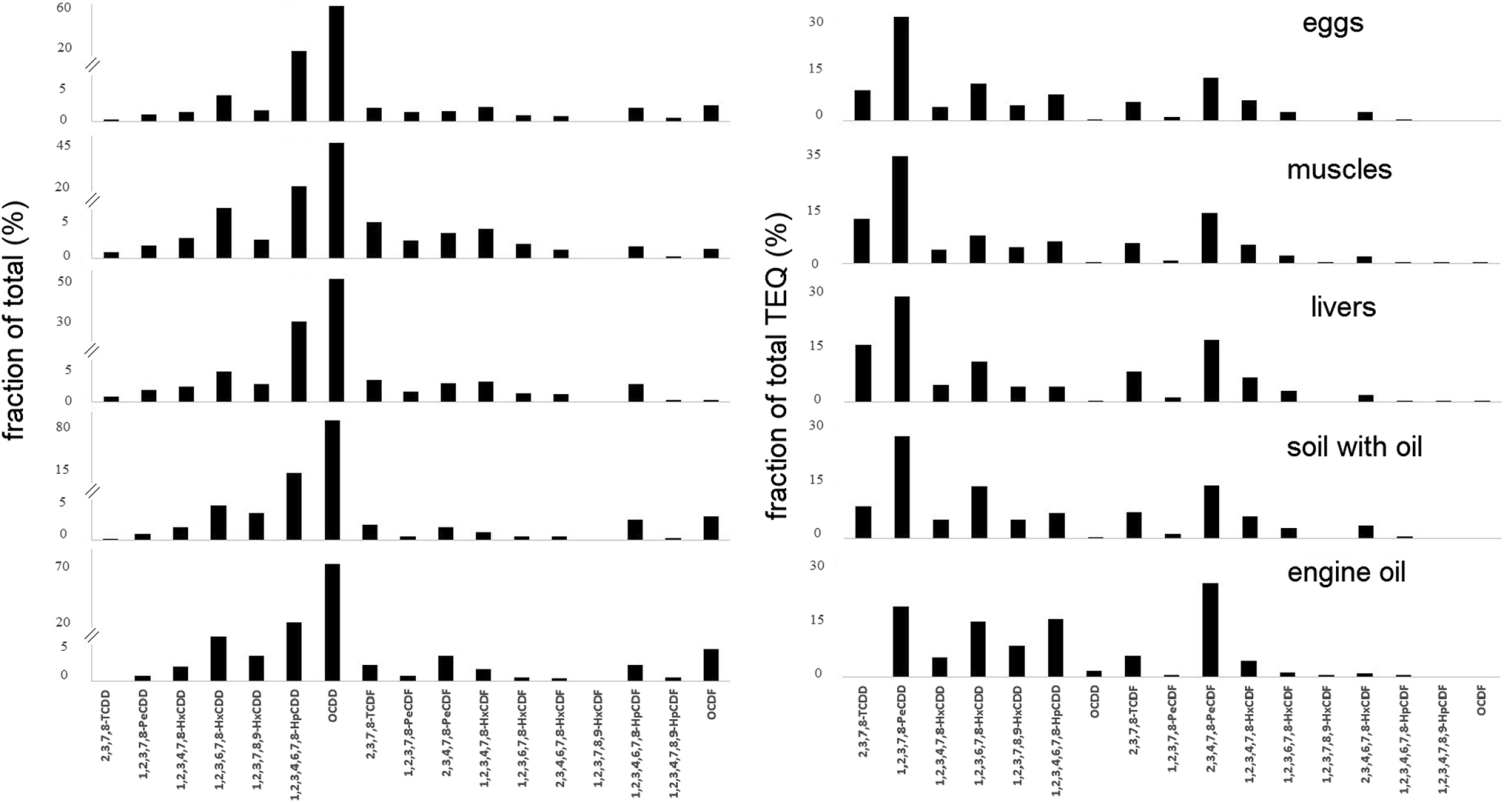
Congener patterns for PCDD/Fs in hens (eggs, muscles, livers), soil with oil and engine oil expressed as a contribution to sum (%) and as a contribution to the TEQ (%)

Congener patterns are an important tool for the identification of sources of PCDD/Fs and PCBs (Hoogenboom et al. 2020). The expression of the patterns has a significant impact on the potential source analysis. Patterns expressed as a relative contribution to the sum are sometimes not enough, and it is necessary to compare the pattern as a relative contribution to the TEQ (Hoogenboom et al. 2020). Figure 1 presents the congener pattern of PCDD/Fs in all the analyzed samples expressed as a fraction of the total percentage and as a fraction of the total TEQ percentage. For the hen eggs (*n* = 3), hen muscles (*n* = 3), and hen livers (*n* = 3), an average profile is presented. In all the analyzed samples, two highly chlorinated PCDD/Fs (1,2,3,4,6,7,8-heptachlorodibenzo-p-dioxin (HpCDD) and 1,2,3,4,6,7,8,9-octachlorodibenzo-p-dioxin (OCDD)) dominated the congener profiles, together contributing 65–92% to the fraction of total PCDD/Fs. The highest contribution to the fraction of total PCDD/Fs was OCDD, 71% in engine oil, 77% in soil (contaminated with oil), and much lower in hen eggs, 57%; livers, 49%; and muscles, 41%. A strong similarity was noticed when comparing the profiles expressed in TEQ. In all the analyzed samples, 1,2,3,7,8-pentachlorodibenzo-p-dioxin (1,2,3,7,8-PeCDD), 1,2,3,6,7,8-hexachlorodibenzo-p-dioxin (1,2,3,6,7,8-HxCDD), and 2,3,4,7,8-pentachlorodibenzofuran (2,3,4,7,8-PeCDF) were the highest contributors to the total TEQ (Fig. 1). In the oil samples, 2,3,4,7,8-PeCDF dominated (24%), while in the hen eggs and tissues, it was 1,2,3,7,8-PeCDD (27–32%). The contamination of the hen tissues by 2,3,7,8-tetrachlorodibenzo-p-dioxin (2,3,7,8-TCDD) came from the soil because this congener was not detected in the engine oil.

According to other researchers (Pirard and De Pauw 2005; Hoogenboom et al. 2006), the fraction of a certain congener excreted in the eggs, in relation to the amount ingested from the feed, is around 40% for lower chlorinated congeners and lower for hepta and octa congeners. Higher chlorinated congers are excreted to a greater extent (Pirard and De Pauw 2005).

**Fig. 2:**
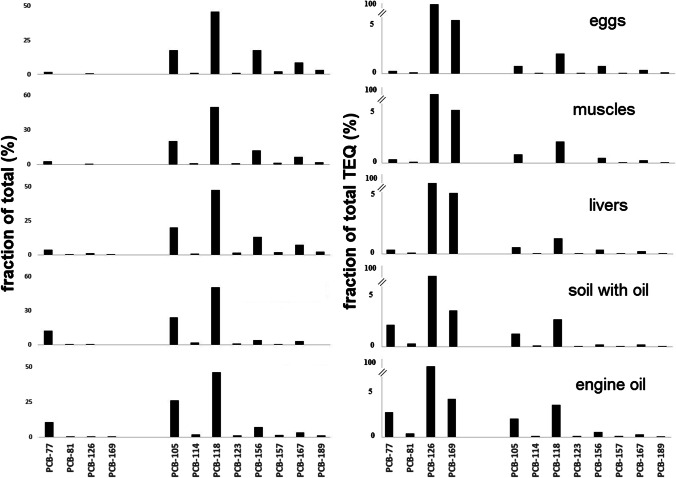
Congener patterns for DL-PCBs in hens (eggs, muscles, livers), soil with oil and engine oil expressed as a contribution to the sum (%) and as a contribution to TEQ (%)

As the PCDD/F patterns were similar, the DL-PCB profiles were also compared (Fig. 2) and showed similarity with the higher contributor to the fraction of total %—PCB-118 (46–50%), PCB-105 (18–26%), and PCB-156 but only in the hen eggs and tissues (12–17%). It contributed to the soil and oil to a much lesser extent (4–7%). For the DL-PCBs, in almost all cases, the contribution of PCB-126 to the TEQ is more than 90% (Hoogenboom et al. 2020). In our case, PCB-126 contributed 91–93% to all matrices, due to it having the highest TEF value of all the DL-PCBs at 0.1. The second largest contributor was PCB-169 (TEF 0.03), at around 3% in the engine oil and contaminated soil, and slightly higher (4–5%) in the eggs and hen tissues.

**Fig. 3:**
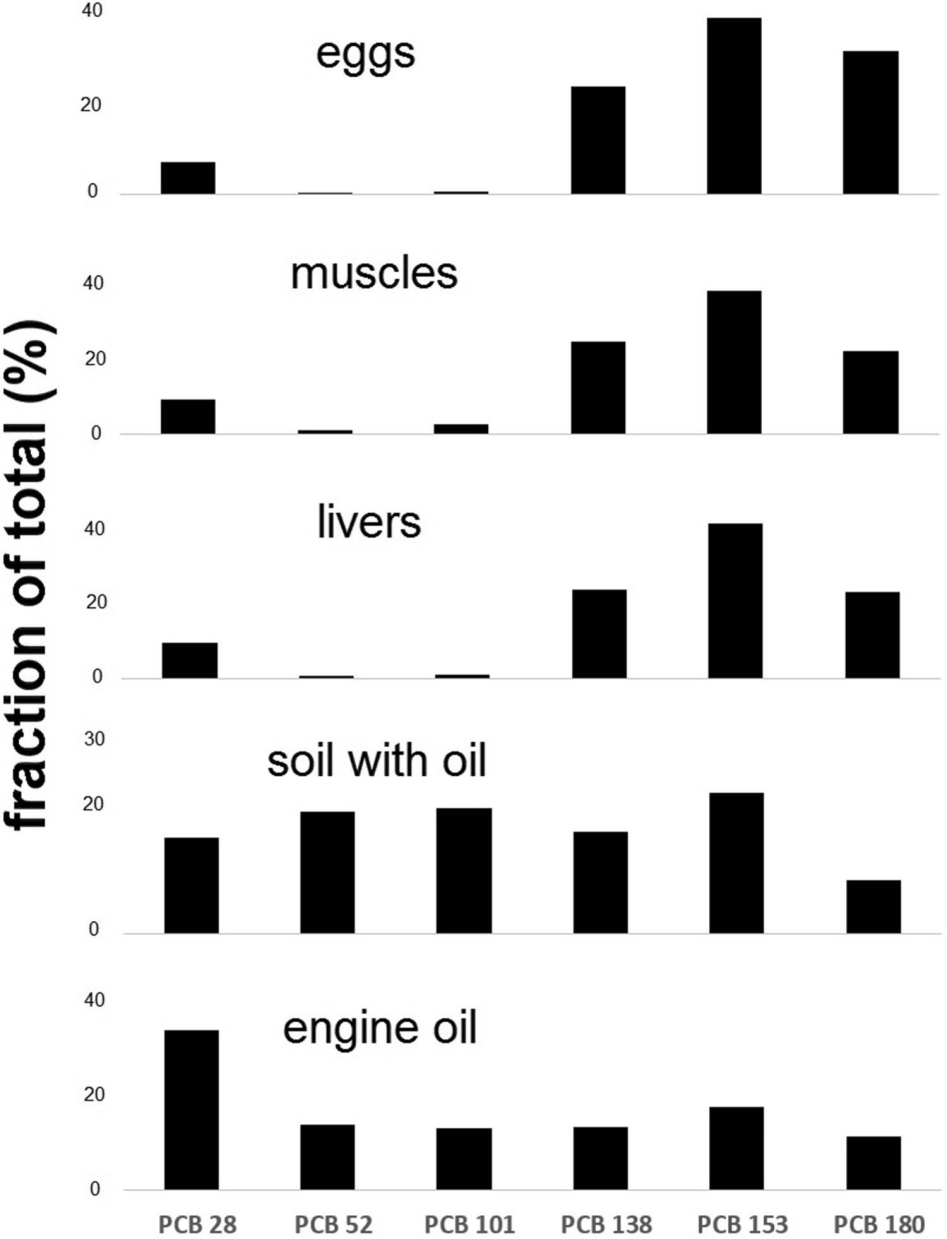
Congener patterns for NDL-PCBs in hens (eggs, muscles, livers), soil with oil and engine oil expressed as a contribution to the sum (%)

For NDL-PCB, the congener profiles are similar for chicken eggs, muscles, and livers (Fig. 3) with PCB-153, PCB-138 and PCB-180 being dominant. PCB-28 is noticeable but makes a smaller contribution (7–10%). However, the profile looks different in the engine oil and oil-soil samples. Apart from PCB-153, PCB-138, and PCB-180, other congeners also contribute significantly (PCB-52 and PCB-101—13–20%). PCB-52 and PCB-101 were practically absent from the chicken eggs, muscles, and livers, but these two congeners have very low bioavailability, which was proved by Hoogenboom (Hoogenboom et al. 2004). The bioconcentration factors (BCFs) for PCB-52, PCB-101, and PCB-28 were 0.5, 0.6, and 1.1, respectively, while for the higher chlorinated congeners 138, 153, and 180, they were about 2.1 (Hoogenboom et al. 2004).

Based on the similarity of the PCDD/F, DL-PCBs, and NDL-PCBS congener profiles, and considering the bioavailability of the individual congeners, it can be concluded that the source of the PCDD/Fs and PCBs in the hens is contaminated oil from the agricultural tractor engine. The results of our work show how important it is to control the environment that free-range hens have access to due to the possibility of the emergence of new sources of pollution.

### Dietary intake—risk for consumers

The dietary intake was calculated for an adult of 70 kg and a child of 23.1 kg (EFSA, 2012) consuming a statistically average number of eggs and an assumed meat portion of 100 g and 200 g for children and adults, respectively. The average monthly consumption of eggs per capita in Polish households in 2019 was 10.99 (equal to around 2.7/week) (Statistical Yearbook 2020). An average egg weight of 60 g was assumed. The calculated intake values, based on consuming this amount and the levels of PCDD/F/DL-PCB found in the eggs and muscles from the contaminated farm, are presented in the Table 2.

**Table 2:** Estimated intake of PCDD/F and DL-PCB expressed as % of TWI

	PCDD/F/DL-PCB concentration	Children	Adult
		% TWI	
Egg 1	4.67	201	66
Egg 2	4.27	180	59
Egg 3	5.61	216	71
Muscles 1	3.85	13	8
Muscles 2	4.07	25	17
Muscles 3	5.46	41	27

To characterize the potential health risk associated with the intake of PCDD/Fs and DL-PCBs, the doses ingested with the hen eggs and muscles were expressed as a percentage of the tolerable weekly intake (TWI) (2 pg WHO-TEQ kg^−1^ body weight) (EFSA 2018).

It was found that, for children, consumption of each of the analyzed eggs results in the TWI being exceeded approximately twice over (Table 2). For adults, the results are lower due to the higher body mass, amounting to around 60–70% of TWI. Consumption of the assumed quantity of chicken meat will result in a PCDD/F/DL-PCB intake at a low level of around 8–27% of TWI and 13–41% of TWI for adults and children, respectively. The results indicate that eggs from the contaminated farm could be a significant source of PCDD/F/DL-PCB. One should keep in mind that this calculation does not take into account other consumed food, which can also contain PCDD/F/DL-PCB, such as fish and milk (Piskorska-Pliszczynska et al. 2017a; Mikolajczyk et al. 2021). The presented case is an anomaly, and the dietary intake calculated here should not be applied to the general population. These results are intended to show that frequent consumption of eggs from a contaminated farm may pose a health concern. Previous papers have proved that eggs from free-range or organic, compared to cage production, are a bigger source of POPs (Roszko et al. 2014; Pajurek et al. 2019; Mikolajczyk et al. 2022). Data from various parts of the world indicates that free-range eggs contribute significantly to the PCDD/F/DL-PCB dietary intake (Hsu et al. 2010; Van Overmeire et al. 2011; Lambiase et al. 2017; Weber et al. 2018; Kudryavtseva et al. 2020; Petrlik et al. 2022).

## Conclusions

Hen eggs are one of the most important components of the human diet. Eggs from home production are becoming increasingly popular among consumers; however, access of hens to the environment is associated with a greater risk of POP contamination. The main conclusions of the study are as follows:

- The results of our research reveal another new potential source of contamination in free-range hen eggs.
- This case shows how important it is to draw attention to chicken paddocks.
- Consumption of contaminated eggs from the described case may cause the TWI to be exceeded for children and contribute significantly to the TWI for adults.
- Analysis of the profiles of dioxin and PCB congeners provides an opportunity to identify sources of contamination.
- Food of animal origin must be controlled to eliminate health risks and protect consumer safety.

The issue of contamination of free-range or organic eggs is a complex problem, and to be able to look at it from a broader perspective, a large group of POPs should be analyzed (chlorinated dioxins, PCBs, polybrominated diphenyl ethers, brominated dioxins, and perfluoroalkyl substances) in egg samples. Such data will allow the assessment of their occurrence in such commonly consumed products and the assessment of human exposure.

## Data Availability

Not applicable.
